# Policy discourse on AMR in food-producing animals: examining framing and language for effective communication

**DOI:** 10.1093/jacamr/dlaf113

**Published:** 2025-07-10

**Authors:** Carly Ching, Muhammad H Zaman, Veronika J Wirtz

**Affiliations:** Department of Biomedical Engineering, Boston University, Boston, MA, USA; Department of Biomedical Engineering, Boston University, Boston, MA, USA; Center on Forced Displacement, Boston University, Boston, MA, USA; Department of Global Health, Boston University School of Public Health, Boston, MA, USA

## Abstract

**Background:**

Indiscriminate use of antimicrobials in food-producing animals is a critical driver of antimicrobial resistance (AMR). However, pushback from stakeholders on policies and regulations on antimicrobial use in food-producing animals remains. One important strategy to promote behavioural change is effective communication. Framing, or how issues are constructed to relate to specific interests, is a mechanism to guide sentiment, including of political stakeholders and end-users.

**Methods:**

Through a sector-specific approach, we used a combination of inductive and deductive coding to quantitatively determine how risk and rationale for action were framed within portions of policy documents and reports from international organizations focused on food-producing animals and AMR. We also qualitatively examined the frames and language used within the documents, to identify specific narratives used, as well as gaps and opportunities to improve communication for end-user support and political legitimacy.

**Results:**

We found that while similar motivational frames are used throughout, they were distributed differently and utilized different narratives. The most frequently used motivational frame, on average, was ‘Human Health’ (20.9% of all frames used) with ‘Animal Health and Welfare’ and ‘Food Production and Security’ second and third, respectively (18.2% and 14.5%). Self-interest frames specific to the farmer or farm worker were rarely used.

**Conclusions:**

Specific recommendations include increasing self-interest frames, ensuring accessibility of messaging and considering underlying assumptions. Overall, our findings can improve framing and language to improve resonance on policies surrounding antimicrobial use in food-producing animals. This work provides a framework to systematically analyse framing in documents to compare different sectors or regions.

## Introduction

Antimicrobial resistance (AMR) is a global public health problem. Specifically, indiscriminate use of antimicrobials in animals is a critical driver of AMR, propelled by high use in the production of food-producing animals.^1^ Policies and uptake of proposed regulations on antimicrobial use in food-producing animals face continued pushback from stakeholders including governments and food producers.^2^ One important strategy to incentivize and nudge behavioural change is effective communication. Emphasizing this point, the Quadripartite One Health Priority Research Agenda for Antimicrobial Resistance^3^ identified the following research priority question: ‘How can information design sciences (presenting information in an accessible and understandable way) be leveraged to improve effective understanding of the information across different stakeholders in the One Health AMR field?’, while Objective 1 of the World Health Organization (WHO) Global Action Plan seeks to ‘Improve awareness and understanding of antimicrobial resistance through effective communication, education and training’.^4^

Framing is a mechanism to guide sentiment and influence viewpoints, including political stakeholders and end-users. Frames refer to ‘an active social construct developed by groups that are deliberately and strategically seeking to convince others of their understanding of an issue and the particular modes of action required to address it’.^5^ More specifically, in 1988, Benford and Snow defined motivational framing as ‘the elaboration of a call to arms or rationale for action that goes beyond the diagnosis and prognosis’.^6^ There is limited research on framing by external agencies and within countries on the issue of AMR. Currently, a few studies have analysed higher-level frames used within policy documents. Wernli *et al*.^7^ identified global health frames relevant to AMR in policy documents using a deductive approach based on frames from previous global health studies. Khan *et al.*^8^ compared framing of AMR in policy documents from global and high-income (HI) countries with that in low-and-middle income countries (LMICs) using deductive coding considering the frames found by Wernli *et al.*^7^ along with new inductive codes. Overall, the authors found conflicting narratives between international-level and HI countries and LMICs, in that LMIC documents framed AMR as a health equity issue more than the international and HI documents. Neither of these two studies provided a quantitative analysis.

Notably, more research is needed to improve communication about AMR and stewardship in animal agriculture;^9^ an outstanding question remains about the language and frames used within each sector (e.g. human, animal, environment). AMR is a One Health problem, in which human, animal and environmental health are interdependent domains.^10^ However, even though multisectoral approaches are favoured in the One Health context, conceptions of One Health largely remain anthropocentric, with a focus on human health.^11^ As such, broad messaging may be ineffective for specific stakeholders central to animal and environmental sectors, as each sector also has different motivating factors and contexts. For example, language and narratives surrounding efforts on behaviour of farmers and farm workers requires understanding of specific cultural contexts, norms and practices, which do not always translate from the human healthcare setting.^12^ In this study, we seek to determine more granular motivational frames related to usage in food-producing animals, how these reflect upon conceptions of One Health, and assess effectiveness of messaging to a specific stakeholder (farm or farm workers).

Thoughtful examination of language used and how frames are received, especially by target groups, can drive effective messaging of campaigns and acceptability of proposed policy. The aim of this study was to identify which motivational frames are used most frequently within policy documents and reports from international organizations within text focused on food-producing animals (specifically poultry and livestock), using a sector-specific approach. Specifically, we use a combination of inductive and deductive coding to analyse global Quadripartite Joint Secretariat on AMR (QJS) policy documents and reports. We qualitatively examine the frames and language used within the documents, to identify narratives used, as well as gaps and opportunities to improve communication. We also quantitatively analyse the frequency of how often the frames are used. We also seek to determine whether, from the viewpoint of the farmer and farm worker, self-interest or public-interest frames are more predominant, to improve targeted messaging. For this study we consider farmers and farm workers the ‘end-user’ as they are the final point in the chain of supply of antimicrobials used in animals. However, we note that farm or farm workers are not the only ones responsible for rational antibiotic use, as decisions have multiple influences.^13^ Overall, we aim to determine effective framing and communication strategies for both end-user support and political legitimacy.

## Methods

We chose recent key policy documents and reports related to AMR published by the global QJS. The QJS represents the WHO, the United Nations Environmental Programme (UNEP), the World Organization for Animal Health (WOAH) and the Food and Agricultural Organization (FAO). Documents from the Global Leaders Group on AMR (GLG), whose secretariat support is provided by the QJS, were also included. The details of documents are provided in Table 1. The documents are referred to by their publishing organization, with the two GLG documents referred to as GLG Report and GLG Note for the Report and Information Note, respectively.

**Table 1.** Documents included in analyses

Document	Published by	Purpose	Total number of sentences in relevant sections (lines)
Global Action Plan (GAP) on Antimicrobial Resistance (2015)^4^	WHO	Provides a global framework to address AMR, with five main objectives.	180
FAO Action Plan on Antimicrobial Resistance 2021–2025 (2021)^14^	FAO	Outlines the FAO Action Plan on Antimicrobial Resistance 2021–2025 to address AMR in food and agriculture sectors, serving as a guide for Members to build capacity.	261
UNEP Bracing for Superbugs (2023)^15^	UNEP	Report provides evidence that the environment plays a key role in the development, transmission and spread of AMR. The report synthesizes current knowledge gaps, and it shows that while several actions are ongoing, more needs to be done, and offers solutions to prevent and respond to AMR.	756
WOAH Strategy on Antimicrobial Resistance and the Prudent Use of Antimicrobial (2021)^16^	WOAH	Recognizing a ‘One Health’ approach, the strategy outlines the goals and tactics to support Members and to encourage national ownership and implementation.	153
GLG on AMR Report—Towards Specific Action in the Response to Antimicrobial Resistance (2024)^17^	GLG	Recommendations for consideration by UN Member States in the outcome document of the High-level Meeting on AMR in September 2024	199
GLG Information Note—Animal Health and Welfare and Antimicrobial Resistance and Use (2022)^18^	GLG	Information Note of the Global Leaders Group	71

To determine motivational frames used (Table 2) and build a consensus coding strategy, two researchers (C.C. and V.J.W.) determined a set of consensus deductive codes (designated by footnote a in Table 3, adapted from Khan and Wernli^7,8^) used in selected portions of text pertaining to AMR and food-producing animals, livestock and agriculture (specific references to aquaculture, companion animals and wildlife, as well as crop production, were excluded) identified from a rapid review of the study documents. In addition, the two researchers inductively extracted frames (codes) for motivation (Table 2). Discrepancies were discussed between the two researchers until agreement between consensus was achieved. The number of individual codes were listed, and thematically similar codes were amalgamated. Novel or conceptually unique inductive codes for motivational framing were then added to the initial list of deductive codes (Table 3) and definitions were refined. Furthermore, motivational frames (Table 3) were categorized as self-interest (to farmer/farm worker) and/or public interest (collective) (Table 2). One researcher (C.C.) then performed line-by-line coding of text specific to AMR or antimicrobial use in animals, livestock or agriculture throughout the whole document. This includes sector-specific sections, lines specifically containing the terms ‘animal’, ‘livestock’ or ‘agriculture’, and sections of text that directly relate back to AMR infections, AMR development or antimicrobial use in animals, livestock or agriculture (File S1, available as Supplementary data at *JAC-AMR* Online). Technical details of specific activities, results and annexes were excluded. Within these lines, we determined if any of the defined codes applied. A second researcher (V.J.W.) performed coding on a subset of documents using a Microsoft Excel file with identified lines and coding system embedded. Iteratively, definitions were refined, and additional inductive codes were added until a consensus was reached. Intercoder reliability was achieved through consensus. To further analyse how the motivational frames are used, lines were grouped by specific narratives. Language and frames used in the context of previous literature, farmer or farm worker support and political legitimacy were examined. To calculate the percentage usage of a frame, we normalized the calculations relative to all motivational frames used within relevant text (i.e. number of times specific motivational frame used/sum of all times any motivational frame was used) of that document. Our study follows the consolidated criteria for reporting qualitative research (COREQ) checklist (Table S1).^19^

**Table 2.** Definitions of responsibility, self-interest and public interest

	Definition
Motivational frame	‘The elaboration of a call to arms or rationale for action that goes beyond the diagnosis and prognosis’^6^
Self-interest [reference of end-user (farmer/farm worker)]	Consequences impact the individual; benefit or concern is person’s own interest or advantage/disadvantage
Public-interest (reference of collective)	Consequences impact the public; benefit or concern is for the public/greater population

**Table 3.** Definitions of motivational frames

	Frame	Definition
Public interest	Human health^a^ (collective)	Broad impact on human health of general population (including hospital patients)
	Food safety	Threat to food safety. Includes safe handling, contamination and foodborne illnesses.
	Economic impact^a^ (collective)	Costs or financial losses incurred by populations/governments (collective), including economic development and poverty.
	Equity/justice^a^	Unjust or unfair inequalities such as access to quality healthcare and medicines impeding the ability to achieve the highest level of health for all. For instance, access to a health professional is restricted in many settings and patients instead access antimicrobials directly via sellers, pharmacies and other types of outlets.
	Global health security^a^	Threat to global health, including international spread of infectious disease that includes spread due to travel. Includes use of pandemic language (including COVID-19) but excludes transmission from food.
	Food production and security^a^	Loss of production through AMR or antimicrobials as an essential tool for production. This threatens sustainability of food systems and availability and access to sufficient supplies of safe and nutritious food.
	Environmental pollution	Environmental contamination and pollution due livestock and agricultural practices.
	Innovation challenge^a^	Market failure and lack of new therapeutics and treatment options, opportunity for innovation.
Self interest	Human health (individual)	Specific health impact on human health of farmers or farm workers.
	Economic impact (individual)	Costs or financial losses incurred by individuals, including threat to livelihood [from viewpoint of end-user (farmer/farm worker) of antimicrobials for animals].
Animal interest	Animal health and welfare	Impact on animal health and the well-being of animals (animal welfare) where motivation is to improve animal health.

^a^Set of consensus deductive codes used for these.

## Results and discussion

### Comparison of narratives and motivational frames utilized

**Figure 1.**
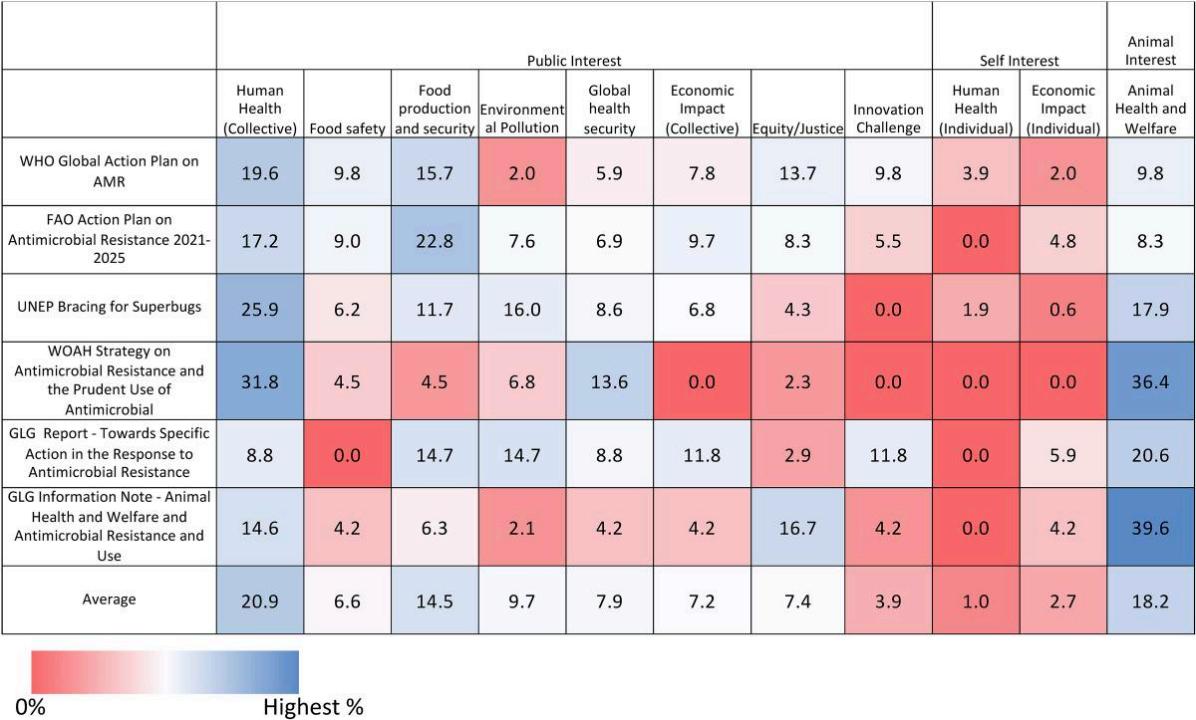
Comparison of motivational frames used. Numbers are normalized to each document and represent frames used as a percentage of all motivational frames used in relevant text.

Within each overarching motivational frame, we identified common and differing narratives and language between documents, summarized in Table 4. Additional example quotes are provided in File S1. Overall, the most frequently used motivational frame, on average, relative to all motivational frames used among all documents included in the study was ‘Human Health (collective)’ (20.9% of all frames used) with ‘Animal Health and Welfare’ and ‘Food Production and Security’ second and third, respectively (18.2% and 14.5%) (Figure 1). The percentage relative to all lines is provided in Figure S1. Among the two most frequently used motivational frames that are specific to animals, ‘Food Production and Security’ and ‘Animal Health and Welfare’, the former represents a risk to the public, while the latter represents a risk to the animal. We found that within the animal sector-specific documents (FAO Action Plan, WOAH Strategy), FAO used the ‘Food Production and Security’ frame the most of all frames (22.8%), while WOAH used the ‘Animal Health and Welfare’ frame the most (36.4%), while having very limited use of the ‘Food Production and Security’ frame (4.5%). This corresponds with the mission of each organization, in that the FAO aims to improve food and agriculture, and the WOAH aims to improve animal health and welfare. While we would expect this imbalance between the WOAH and FAO, we find that the negative relationship between the ‘Animal Health and Welfare’ frame and the ‘Food Production and Security’ frame tends to extend to all documents (Figure S2). In other words, the lower the frequency of the ‘Food Production and Security’ frame the higher the number of ‘Animal Health and Welfare’ frames among all documents. Indeed, ‘Human Health (collective)’ is the second most frequently used frame for the WOAH and FAO documents as opposed to a more animal-specific frame. This suggests that there is often a focus on ‘one or the other’ of the major animal-specific motivational frames; however, without further background information, we cannot speak to the reason why this is. Moreover, the ‘Environmental Pollution’ frame had limited use, demonstrating that within One Health documents, the connection to human health is still privileged, even in sections specific to another sector. This imbalance limits a non-anthropocentric One Health conception. Overall, by quantitatively assessing the motivational frames used we can determine and compare the distributions (Figure S3); using graphical representations, the more balanced a document (i.e. similar percentage use of each frame), the more symmetrical the representation will be, while emphasis on specific frames appear as extended vertices. By comparing these profiles, we observed that the WHO and FAO have similar profiles, while the WOAH and the GLG Note are more like each other, although with WOAH focusing more on ‘Global Health Security’ and the GLG Note focusing more on ‘Equity and Justice’ (Figure S3).

**Table 4.** Summary of narratives and language used within each motivational frame

	Narratives and language	Quotes
Human health (collective)	Two main health-related narratives of resistant bacterial infections and malnutrition. Broad and unspecific language Overlap with food safety and discussion of contaminated food products.	‘AMR is a global threat, and the emergence of antimicrobial-resistant pathogens threatens decades of progress against infectious diseases in animals and humans.’ (WOAH) ‘This trend is troubling for producers and for patients as a fraction of all drug-resistant infections in humans have also been associated with foodborne or animal sources.’ (FAO)
Food safety	Spread of antibiotic-resistant bacteria across the food chain. However, some explicitly used the term food safety without explanation limited language about safe handling and processing	‘Food is one of the possible vehicles for transmission of resistant bacteria from animals to human beings and human consumption of food carrying antibiotic-resistant bacteria has led to acquisition of antibiotic-resistant infections.’ (WHO) ‘The use of antimicrobial drugs has advanced global public health, animal health, and food safety and security.’ (FAO)
Economic impact (collective):	Broad language, with some expansion on the cause of the economic loss. Limited references to specific numbers and study references, which provide a sense of the magnitude of the impact. Poverty had limited discussion.	‘Antimicrobial resistance is a drain on the global economy with economic losses due to reduced productivity caused by sickness (of both human beings and animals) and higher costs of treatment.’ (WHO) ‘In the next decade, it could result in a gross domestic product (GDP) shortfall of US$3.4 trillion annually and push 24 million more people into extreme poverty (World Bank 2017).’ (UNEP)
Equity and justice	Main narrative was on unequal access to quality medicines, services and oversight, especially in LMICs. Few documents highlighted inequities between certain groups, including women.	‘Existing issues and inequalities regarding national access to veterinary and laboratory services, quality, affordable and standardized antimicrobials, and alternatives to antimicrobials for animal health must be addressed.’ (GLG Note) ‘As women are less likely to be compensated—or compensated at a lower level—than men for their efforts in food production and food preparation (FAO, 2011), there is a disproportionate risk of exposure to resistant pathogens relative to financial compensation, highlighting gender equality issues as well (SDG 5).’ (FAO)
Global health security	Broad references to global health without explanation used. Mostly, language referred to spread across borders through travel to highlight how global spread occurs. Another narrative was COVID-19 and pandemics.	‘Responsible antimicrobial use should be considered an integrated part of global health, protecting the health of humans, animals, plants, and the environment.’ (GLG Note) ‘Drug-resistant bacteria can circulate in populations of human beings and animals, through food, water and the environment, and transmission is influenced by trade, travel and both human and animal migration.’ (WHO) ‘As the COVID-19 pandemic has highlighted, the health of humans, animals and the environment are interlinked’ (GLG Note)
Food production and security	Explicit references to food production and security. Another common narrative was highlighting the increasing demand for food animals.	‘Loss of effectiveness of antimicrobials has a negative impact on both animal health and welfare, as well as a significant impact on livestock production and the global food chain, compromising food security.’ (GLG report) ‘The projected increase in demand for animal food products may lead to yet further increases in antibiotic use.’ (WHO)
Environmental pollution	Similar narrative about contamination from animals and farms was used across documents.	‘Up to 90% of antibiotics administered to livestock are excreted, entering directly into the soil and nearby surface and groundwater.’ (GLG Report)
Innovation challenge	Opportunities for innovation in treatments, alternatives and diagnostics were highlighted.	‘There are opportunities to innovate for safe and efficacious alternatives to antimicrobials for good health and productivity in plants and animals.’ (FAO)
Human health (individual)	Motivational frame specific to human health of livestock worker was limited to the WHO GAP and UNEP. Language was direct and used examples, specifically MRSA	‘For example, farmers working with cattle, pigs and poultry that are infected with methicillin-resistant *Staphylococcus aureus* have a much higher risk of also being colonized or infected with these bacteria.’ (WHO)
Economic impact (individual)	Language focused on losses from production, mentioning livelihoods and profitability.	‘For farmers, animal husbandry and the food industry, the loss of effective antimicrobial agents to treat sick animals damages food production and family livelihoods.’ (WHO) ‘There are opportunities to boost profitability through more effective agriculture practices.’ (FAO)
Animal health and welfare	Some specifically used the term welfare throughout while others used it sparingly. Another less frequently used approach was utilizing language about antimicrobials as a tool to treat sick animals.	‘In intensive animal production systems, antimicrobials are frequently relied upon to maintain livestock health, welfare and productivity, including for control of diseases.’ (UNEP) ‘Antimicrobials have been essential in reducing the burden of infectious disease in humans, animals and plants for decades.’ (UNEP)

**Figure 2.**
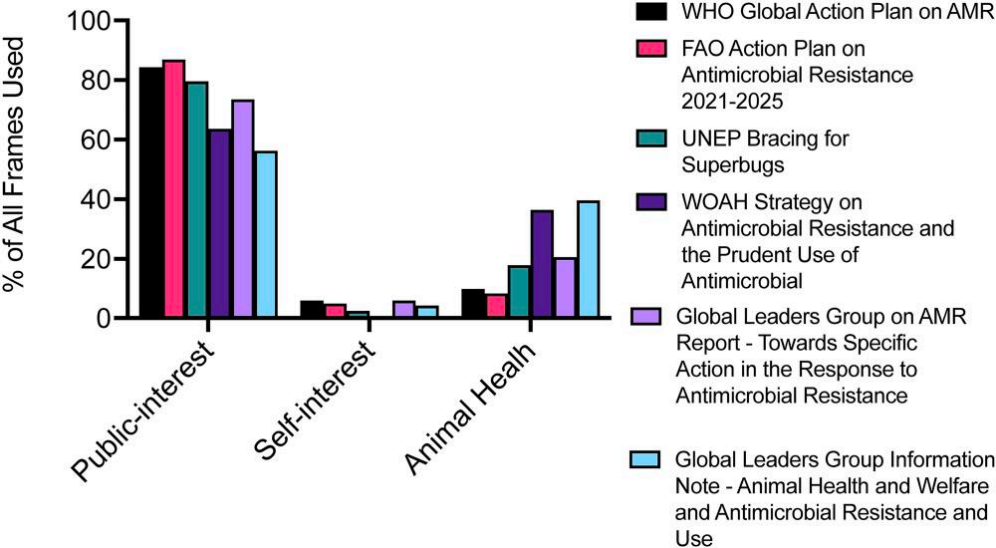
Comparison of public-interest, self-interest and animal health motivational frames used as a percentage of all identified frames used within the documents within relevant text.

Public-interest motivational frames that were not used at all within individual documents were ‘Food Safety’ (GLG Report), ‘Economic Impact (Collective)’ (WOAH) and ‘Innovation Challenge’ (UNEP and WOAH). Cumulatively, self-interest frames for farmers or farm workers were used the least frequently (1.0% for Human Health and 2.7% for Economic Impact). Four out of six documents did not use the individual health frame for farmers or farm workers, including FAO, whose focus is food and agriculture, while the documents all made use of the ‘Human Health (Collective)’ frame. Overall, self-interest frames were used the least compared with public-interest and animal health frames (Figure 2). However, while we have separated specific interests of ‘farmers or farm workers’ (self-interest) from ‘the community’ (public-interest), it is important to note that farmers are themselves part of the broader community and thus act with overlapping agency.

Overall, we found that while similar motivational frames are used throughout, they were distributed differently and, moreover, different narratives were used (Table 4, Figure 1, Figures S2 and S3). While we expect and observe differences based on the mission of each organization, resonance can be improved by having more balanced distribution of frames to allow for broader appeal to multiple stakeholders. After identifying and quantifying the frames used, we qualitatively examined the frames and language within the documents, to identify specific gaps and opportunities to improve communication. Below we expand on these gaps and opportunities to improve framing and communication, how they pertain to end-user support and political legitimacy, and discuss how the results can strengthen the evidence base on effective messaging.

### Increasing personal relevance and self-interest frames can increase resonance

Benford and Snow^20^ describe how effectiveness or resonance of frames is a function of different factors. This includes frame consistency (consistency between actions of organizations and governments and framing), empirical credibility and if the movement framings are congruent or resonant with personal, everyday experiences.^20^ Herein is a central weakness to resonance within AMR communication; as already noted, problems with language describing AMR include terminology and a lack of personal relevance.^21^ It has been suggested by an independent network that current narratives involving blame or superbugs are ineffective and a shift to people-centred perspectives around equity and sustainability is more applicable across sectors.^22^ Further discussion by the Interagency Coordination Group (IACG) on AMR also suggests that language should be connected to the language of everyday living.^23^ In 2019, the Wellcome Trust published guidance on how to effectively communicate about AMR.^24^ The first principle was to frame AMR as undermining modern medicine, which speaks to the recommendation to transition to language that feels more personal, specifically that every day, routine operations are now threatened. However, this framing is focused on human health, and one update is undermining modern medicine and food production systems. Some additional frames specific to use for AMR in food-producing animals could include increases in foodborne illnesses for young children and elderly and pregnant people, the potential health benefits of eating antibiotic-free meat, and examples of infectious disease with economic impact to farmers such as bird flu, which were not found in any of the documents. Coronavirus SARS-CoV-2 (COVID-19) as a frame, used in 3/5 documents published post pandemic, is another important framing device linked to personal experience, representing a recent global lived experience with a zoonotic disease.

Specifically, the idea of linking motivation to everyday living also asks the question of whether most farmers or farm workers have first-hand experience with AMR, or if it is instead not congruent with their everyday experiences and does not feel tangible. How do we address a disconnect between a so-called problem when most farmers or farm workers view antibiotics as an essential tool? Indeed, we found limited use of specific self-interest frames for farmers or farm workers (Figure 2) that build the relationship or applicability to self. MRSA is probably the most well-known example of a livestock-associated resistant bacterial infection:

‘*For example, farmers working with cattle, pigs and poultry that are infected with methicillin-resistant Staphylococcus aureus have a much higher risk of also being colonized or infected with these bacteria.’ (WHO)*

This example highlights well the risk to personal human health of farmers or farm workers, which is an important motivator. However, there were no specific examples that explicitly describe the impact of AMR on the loss of food-producing animals. If there are limited widespread examples, the utility to prevent and treat bacterial infections and loss of an essential tool could be stressed as an alternative. Furthermore, better understanding the motivation for why farmers use antibiotics (to treat, prevent and control disease, to increase productivity, ‘that is just what is done’ etc.) can also help tailor messaging and alternatives. As noted above, while specific interests of ‘farmers’ (self-interest) are discussed separately from ‘the community’ (public-interest), farmers are also part of the broader community with overlapping interests.

A study analysing frames used in mainstream news-media and farming print media in the UK found three distinct frames regarding agricultural use of antibiotics: a system failure frame linking intensive livestock production to AMR in humans; a status quo frame that argues there is limited evidence and stresses some antibiotic use is necessary for animal health; and a third only in farming media, which highlights the need for voluntary and industry-led action in terms of farmer self-interest.^5^ In our analyses of policy documents by global health institutions we also found use of the systems failure frame and references to antibiotics being important tools. However, regarding limited evidence, documents more so discussed the need for more evidence on specific topics and not that evidence does not support the link. Frames for farmer self-interest, however, were minimal (Figure 2). Notably, the self-interest frame was in farming print media and could be assumed to be tailored for this specific audience. Thus, there is a need to increase self-interest frames to convince farmers or farm workers of the benefits of prudent antimicrobial use. A recent study on the use of framing in COVID-19 messaging also found a combination of personal and public-interest frames is more effective that just personal or public-interest frames.^25^

### Assumption of prior knowledge and universal norms undermines accessibility of messaging

Different narratives and approaches were used within overarching frames (Table 4). Moreover, we found many surface-level references to motivational frames without the ‘why’ or specific examples. This could result in some motivational frames being taken at face value, with the consequences not understood or considered seriously. Indeed, it has been noted that prior knowledge and awareness will differ by region, which can lead to confusion regarding narratives pertaining to overuse in humans and animals.^24^ Thus, underlying assumptions of knowledge should be considered to ensure accessibility. We found instances of this throughout. For example, there were mentions of a ‘global health threat’ without expansion on what global health means or why it is a global health threat; some documents use the term but do not include details about how spread is global (i.e. lack of borders, pandemics), while others do not reference a global health threat but mention these reasons. Similarly, for ‘Food Safety’, one document mentioned the term food safety but did not expand on what this means. This is a gap, especially since foodborne diseases are largely how animal health is connected to human health.

Moreover, as mentioned previously, efforts to promote change specific to farmers or farm workers requires focus on specific cultural contexts, norms and practices, which do not always translate from the human healthcare setting.^12^ For example, while human and animal health are grouped together in a One Health approach, the actions for agriculture are much more of a voluntary (in the absence of laws) ask to be better than standard practices compared with human healthcare, where appropriate use of antimicrobials entails adhering to clinical guidelines (the standard). As such, framing everyday normal usage as misuse could confuse and frustrate farm end-users, when it is the norm; using the term misuse to both humans and animals assumes the same norms. Take for example the following lines:

‘*However, overuse and misuse of antimicrobial agents in humans, animals and plants sectors has dramatically accelerated the emergence of AMR.’ (WOAH)*

‘*The overuse and misuse of antimicrobials in animal and plant production is influenced by an interplay of factors.’ (FAO)*

While certain groups and policymakers may view it as misuse, farmers may not. Thus, for animal use, legislation may be required more than in human healthcare for it to be considered misuse.

Furthermore, while our findings are from documents for broad global audiences, the frames and language identified may not be of universal relevance. Indeed, cultural practices and norms will differ between countries. To achieve global governance, countries need to agree to enforce certain standards, and a common roadblock is that many countries view global governance and priorities as expressing a Western viewpoint.^26^ Thus, not considering these differences in framing and language can further division. More inclusive language that does not assign blame is important. Indeed, in many of these documents a hierarchy of best practices is implied between regions. For example:

*‘In some jurisdictions, antimicrobials are still used as growth promoters.’ (UNEP)*

The use of the word still implies that this practice should have already stopped; however, the resources and situation that enable this in the absence of alternatives is very different among different regions and countries. International organizations have an important role in effective framing but need to be careful to avoid a region-specific viewpoint.

### AMR as a threat to food security as an animal-specific motivational frame amenable for political legitimacy and global governance

Among the two most frequently used motivational frames that are specific to animals, ‘Food Production and Security’ and ‘Animal Health and Welfare’, one is targeted towards the collective (food production) and one is intrinsic to animals. While both are important, food security is more accepted as part of domestic and foreign policy compared with animal welfare. There are no official international agreements ensuring animal welfare.^27^ Conversely, there are international treaties and agreements related to food security.^28–30^ This may be because, in terms of foreign policy, food security is of importance to global stability and is also a global issue given the interconnectedness of food systems. However, AMR is not always prominent in food security strategies. For example, the U.S. Government’s Global Food Security Strategy is an integrated whole-of-government approach that aims to end global hunger, poverty and malnutrition through the Feed the Future initiative.^31^ Within the strategy, AMR is mentioned only twice as a description of the mission of one specific office, rather than as part of the strategy. Instead, climate change and conflict are the major drivers that the strategy addresses, which can also promote AMR themselves. Tying AMR to food security ties it to tangible consequences, which include economic growth, community resilience, nutrition and decreased poverty. Thus, emphasis on how AMR and antimicrobial use are part of food security and sustainability could help add legitimacy for global governance and government engagement for inclusion of AMR in food security strategies. Conversely, though, this may perpetuate framing back to human health and again promote a perceived reduced importance of animal health and welfare as an important intrinsic value.

### Motivational frames around global health security to mobilize funds

Global health is another frequently used frame mostly understood as health issues that transcend national border with a focus on security rather than equity or justice-related issues. Literature has described that health security is an important driver to mobilize donors compared with human rights issues. Health security often appeals more to donors due to its immediate and broad implications for global stability and security. This can be seen in the way health emergencies, such as pandemics, are framed as threats to international security, leading to swift and significant funding from international donors.^32^

One of the key drivers behind this donor preference is the framing of health security as a matter of global stability. For instance, affluent countries see the spread of infectious diseases in the regions outside their own influence as a threat to their own population and their governments can justify spending towards their domestic audiences and voters as a security matter.^33^ This framing makes it easier to mobilize resources from countries that might be less responsive to appeals based solely on human rights, which are often perceived as more localized or less urgent.

On the other hand, while equity and justice issues such as access to healthcare are fundamentally important, they tend to mobilize a different set of donors who are more focused on long-term development and equity issues rather than immediate security threats (e.g. philanthropic donors). Human rights-based approaches emphasize equity, justice and the protection of vulnerable populations, which are crucial but often require a different advocacy strategy to garner support.

### Limitations

In this study, we examined the motivational and diagnostic frames used to discuss AMR in the food-producing animal sector. Some limitations to our work include the restricted set of documents analysed, which were defined and published by a network of connected agencies. While this could introduce bias, we found that the distribution of frames varied among the documents (Figures S2 and 3). As independent organizations are performing a lot of research and work in antibiotic stewardship, we included grey literature in our discussion. While such reports are informed by experts and evidence, they may also be biased or reflect the interests of specific groups. We also note that while the selected documents are landmark reports representative of AMR global policy documents, they may miss critical regional nuances and frames. With our focused approach targeted to food-producing animals and end-users, we made broad assumptions in classifying frames as public-interest, when there will be overlapping interests, and independent interests within individual frames as well. For example, the intrinsic interests of the environment compared with how it impacts the community and environmental stakeholders. Thus, while the analyses provided here are limited to one sector and international organizations, this work also provides the foundation for a framework to systematically analyse documents, including communication materials, campaigns and national action plans, to compare framing within different sectors or regions. These different sectors include the environment and human health.

### Recommendations

Overall, our findings can improve framing and language to improve resonance and uptake of activities and policies to restrict or alter behaviours surrounding antimicrobial use in food-producing animals. Specific recommendations include increasing personal relevance and self-interest frames as they pertain to the end-user, thinking carefully about the accessibility of messaging and underlying assumptions, and identifying frames that are most amenable to global governance and political legitimacy, of which we provide suggestions above. While our work is sector-specific, and specific to reduction of irrational antibiotic use in food-producing animals, as can be seen from our analysis the focus within One Health approaches is skewed towards human health, rather than the environment, even though it is a tripartite model. Thus, a conscious effort still needs to be made to present and uphold an integrated perspective of all domains. We note, however, that recommendations will likely be context- and audience-specific and require further feedback from multiple stakeholders, including veterinarians, drug sellers, communication experts and regulatory authorities and the specific end-user population. As such, future research should include better understanding the motivation for farmers or farm workers to use antibiotics, to improve targeted messaging and provide alternatives. Furthermore, work needs to be done to determine effective strategies of how messages are being delivered, including understanding community structure and who can effectively provide information and guidance.

## Supplementary Material

dlaf113_Supplementary_Data

## Supplementary data

File S1, Figures S1 and S2 and Table S1 are available as Supplementary data at *JAC-AMR* Online.
